# Divergent Cardiac Effects of Angiotensin II and Isoproterenol Following Juvenile Exposure to Doxorubicin

**DOI:** 10.3389/fcvm.2022.742193

**Published:** 2022-03-25

**Authors:** Kevin Agostinucci, Marianne K. O. Grant, Davis Seelig, Doğacan Yücel, Jop van Berlo, Alessandro Bartolomucci, Jason R. B. Dyck, Beshay N. Zordoky

**Affiliations:** ^1^Department of Experimental and Clinical Pharmacology, University of Minnesota College of Pharmacy, Minneapolis, MN, United States; ^2^Department of Veterinary Clinical Sciences, University of Minnesota College of Veterinary Medicine, St. Paul, MN, United States; ^3^Department of Integrative Biology and Physiology, University of Minnesota Medical School, Minneapolis, MN, United States; ^4^Department of Medicine, Lillehei Heart Institute, University of Minnesota Medical School, Minneapolis, MN, United States; ^5^Department of Pediatrics, Faculty of Medicine and Dentistry, Cardiovascular Research Centre, University of Alberta, Edmonton, AB, Canada

**Keywords:** anthracycline-induced cardiotoxicity, doxorubicin, angiotensin II, hypertension, isoproterenol

## Abstract

Hypertension is the most significant risk factor for heart failure in doxorubicin (DOX)-treated childhood cancer survivors. We previously developed a two-hit mouse model of juvenile DOX-induced latent cardiotoxicity that is exacerbated by adult-onset angiotensin II (ANGII)-induced hypertension. It is still not known how juvenile DOX-induced latent cardiotoxicity would predispose the heart to pathologic stimuli that do not cause hypertension. Our main objective is to determine the cardiac effects of ANGII (a hypertensive pathologic stimulus) and isoproterenol (ISO, a non-hypertensive pathologic stimulus) in adult mice pre-exposed to DOX as juveniles. Five-week-old male C57BL/6N mice were administered DOX (4 mg/kg/week) or saline for 3 weeks and then allowed to recover for 5 weeks. Thereafter, mice were administered either ANGII (1.4 mg/kg/day) or ISO (10 mg/kg/day) for 14 days. Juvenile exposure to DOX abrogated the hypertrophic response to both ANGII and ISO, while it failed to correct ANGII- and ISO-induced upregulation in the hypertrophic markers, ANP and BNP. ANGII, but not ISO, worsened cardiac function and exacerbated cardiac fibrosis in DOX-exposed mice as measured by echocardiography and histopathology, respectively. The adverse cardiac remodeling in the DOX/ANGII group was associated with a marked upregulation in several inflammatory and fibrotic markers and altered expression of *Ace*, a critical enzyme in the RAAS. In conclusion, juvenile exposure to DOX causes latent cardiotoxicity that predisposes the heart to a hypertensive pathologic stimulus (ANGII) more than a non-hypertensive stimulus (ISO), mirroring the clinical scenario of worse cardiovascular outcome in hypertensive childhood cancer survivors.

## Introduction

The survival rate of childhood cancer has increased from 60% to more than 85%, thanks to advanced diagnosis, treatment, and care models (1). Indeed, there are more than 500,000 childhood cancer survivors in the United States and this number is expected to increase. Although the increased survivorship is a cause for celebration, up to 73% of childhood cancer survivors suffer from long-term health complications (2). Cardiovascular disease is one of the most common long-term complications in survivors and the second leading cause of death in childhood cancer survivors after secondary malignancy (2). The high burden of cardiovascular diseases in childhood cancer survivors is mainly attributed to cardiotoxic cancer treatments such as anthracyclines and radiation therapy (3). Doxorubicin (DOX) is an anthracycline chemotherapeutic agent widely used in the treatment of lymphoma, leukemia, and other pediatric cancers, despite its known cardiotoxic effects (4). Since the severe cardiotoxic effects of DOX are dependent on the cumulative dose, the current treatment protocols usually do not exceed this threshold. Therefore, the rates of severe cardiovascular complications have declined in recent years. However, it has also been shown that low cumulative doses of DOX cause subclinical cardiotoxicity in childhood cancer survivors (5–7).

DOX-induced subclinical cardiotoxicity predisposes the survivors to adult-onset cardiovascular risk factors in a two-hit manner (8, 9). Given the expected long survivorship life in childhood cancer survivors, many of them would develop multiple cardiovascular risk factors later in their adult life, which can be considered as “second hits.” Since hypertension is the most significant cardiovascular risk factor for all adverse cardiac events, including heart failure and cardiac death, in anthracycline-treated childhood cancer survivors (10), we have recently developed a two-hit mouse model of juvenile DOX-induced latent cardiotoxicity that is exacerbated by adult-onset angiotensin II (ANGII)-induced hypertension (11). Nevertheless, it is still not known how juvenile DOX-induced latent cardiotoxicity would predispose the heart to other cardiovascular pathologic stimuli that do not cause hypertension. In the current study, we characterize the detrimental synergy in the DOX/ANGII model in parallel to a new model wherein juvenile DOX exposure is followed by adult-onset catecholamine stress by daily injections of isoproterenol (ISO). ISO is a non-specific beta-adrenoceptor agonist that is commonly used to induce a dose-dependent cardiac pathology without elevating blood pressure (12–15). Characterizing both DOX/ANGII and DOX/ISO models is critical to understanding why hypertension is the most significant risk factor for cardiovascular morbidity and mortality in anthracycline-treated childhood cancer survivors and thereby devising effective therapeutic strategies against this significant clinical problem.

## Materials and Methods

### Animals

**Figure 1:**
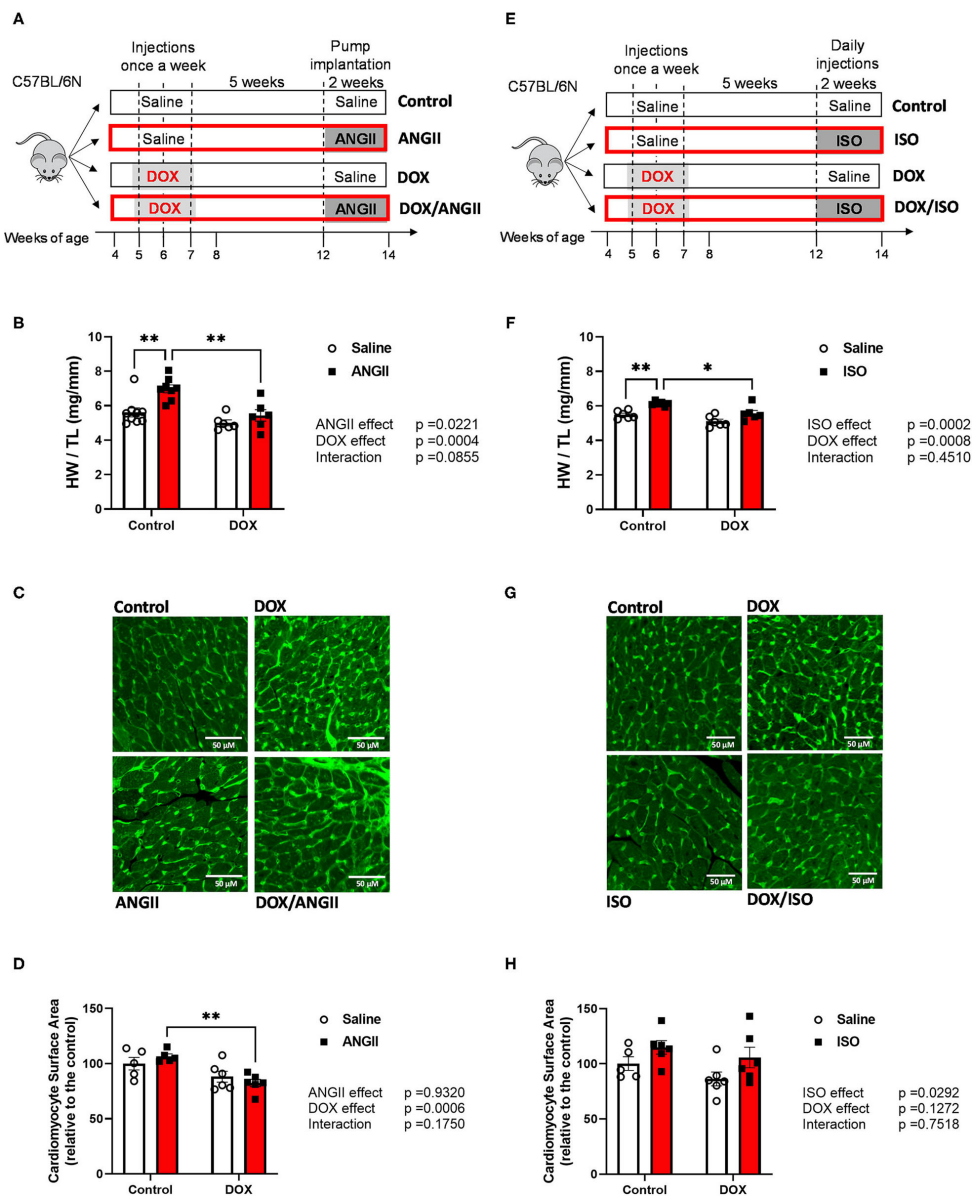
Experimental design of the two-hit models of latent DOX cardiotoxicity using ANGII **(A)** or ISO **(E)** as second hits. Male 5-week old mice were administered DOX (4 mg/kg/week) or saline for 3 weeks and allowed to recover for 5 weeks prior to exposure to ANGII infusion (1.4 mg/kg/day for 14 days) or ISO injections (10 mg/kg/day for 14 days). Hypertrophic response to ANGII and ISO is abrogated by juvenile exposure to DOX. **(B,F)** Heart weight to tibial length ratio (HW/TL) (*n* = 6-9 per group). **(C,G)** Representative heart sections. **(D,H)** Quantification of cardiomyocyte surface area; bar scale = 50μM. Values are represented as means ± SEM. Statistical significance of pairwise comparisons was determined by two-way ANOVA with Tukey's *post-hoc* analysis (*p < 0.05, **p < 0.01). ANGII, Angiotensin II; DOX, doxorubicin; ISO, isoproterenol.

Animal procedures were approved by the Institutional Animal Care and Use Committee (IACUC) at the University of Minnesota (Protocol ID: 1807-36187A). Animal housing and all animal procedures were performed at the University of Minnesota according to the approved protocol. Male 4-week old C57BL/6N mice were purchased from Charles River Laboratories. All mice were housed in groups of 3-4 mice per cage, maintained under standard specific pathogen free (SPF) conditions, and given food and water *ad libitum* in a 14 h light/10 h dark cycle and at 21 ± 2°C. After a 1-week acclimation period, 5-week old mice were administered either DOX (4 mg/kg/week for 3 weeks, DOX group) or equivalent volume of sterile normal saline (control group). The mice were monitored twice per week and were weighed once weekly. Five-weeks following the last DOX injection at the age of 12 weeks (the age of young adult mice), control and DOX-treated mice were assigned to either the ANGII or ISO experiments. In the ANGII experiment (Figure 1A), control and DOX-exposed mice were infused with ANGII (1.4 mg/kg/day) or sterile normal saline for 14 days through subcutaneously implanted ALZET osmotic mini-pumps (Durect Corp, Cupertino, CA) to induce hypertension as previously reported (11, 16, 17). Animals were anesthetized with isoflurane (2-3%) and surgical site was clipped then cleaned with betadine and alcohol. Anesthetic level was assessed by toe pinch and respiratory rate. A skin incision was made with surgical scissors in the mid-scapular area, a filled pump was inserted into the pocket, and the wound was closed with skin staples. For analgesia, animals were administered carprofen (5 mg/kg) just prior to the surgery and daily for 3 days following surgery and monitored for any signs of infection or suture opening. In the ISO experiment (Figure 1E), 10 mg/kg ISO or an equivalent volume of sterile normal saline was administered by subcutaneous daily injection for 14 days as previously reported (12). At the end of the experiment, mice were humanely euthanized by decapitation under isoflurane anesthesia and hearts were harvested.

### Echocardiography

All heart function and wall thickness data was measured using echocardiography. Baseline cardiac function was assessed 5 weeks after the last DOX treatment on the day prior to the start of the 14 day ANGII or ISO challenge. To determine the response to prolonged ANGII administration, cardiac function was assessed by echocardiography on the 15th day after implanting the mini-osmotic pumps containing either saline or ANGII in control and DOX-treated mice (*n* = 6-9 per group). To determine the response to ISO administration, cardiac function was assessed by echocardiography 24 h following the last dose of ISO or sterile saline injections in control and DOX-treated mice (*n* = 6 per group). Echocardiography was performed using the Vevo 2100 system (VisualSonics, Inc., Toronto, Ontario, Canada) equipped with an MS400 transducer. Anesthesia was induced with 3% isoflurane in oxygen and maintained at 1-2% during the procedure. Mice were secured in a supine position on a heated physiologic monitoring stage. Parasternal short axis images of the left ventricle were obtained in M-Mode at the level of the papillary muscles. Endocardial and epicardial borders were manually traced over three cardiac cycles and measures of cardiac function and morphometry were calculated using VisualSonics cardiac measurement package of the Vevo 2100.

### Histopathology

Left ventricular (LV) heart sections were collected, fixed in 10% neutral buffered formalin and embedded in paraffin. Four-micron sections were stained with hematoxylin and eosin (H&E) or Masson's trichrome stain. Histopathologic evaluation was performed by a board-certified veterinary pathologist who was blinded to the experimental group. Inflammation and fibrosis were assessed as follows: 0, absent; 1, minimal inflammation or fibrosis; 2, mild inflammation or fibrosis; 3, moderate inflammation or fibrosis; and 4, marked inflammation or fibrosis. Sections from each heart were also immunohistochemically stained for expression of MAC-2 (galectin-3). In brief, four-micron sections were dewaxed and rehydrated prior to antigen retrieval. Thereafter, sections were incubated with anti-galectin-3 antibody (clone M3/38, Cedarlane Labs, Burlington, NC) according to manufacturer's instruction. The number of MAC-2 positive cells was manually quantified on the five most cellular 200X images. To measure cardiomyocyte cross-sectional surface area from histological sections, we stained dewaxed and rehydrated sections with Fluorescein isothiocyanate-conjugated wheat germ agglutinin (5 μg/ml, Vector Laboratories FL-1021) and 4′,6-diamidino-2-phenylindole (DAPI, Invitrogen D3571). Stained slides were mounted with Vectashield (Vector Laboratories H-1000). Images were acquired using a Nikon TiE or a Zeiss Axio Images M1 microscope, both equipped with a digital black/white camera. Wheat germ agglutinin binds to glycosylated proteins, which are enriched in the membranes of cells. Based on the difference in size between cardiomyocytes and non-cardiomyocytes, we traced the area of cardiomyocytes using Image J. We selected areas where cardiomyocytes had a round shape, indicative of a cross-sectioned cardiomyocytes. We traced at least 100 cardiomyocytes per heart in different areas of a cross-sectioned heart. Images were quantified by a researcher blinded to the treatment.

### RNA Extraction and Real-Time PCR

Total RNA was extracted from 20 mg frozen heart tissue using 300 μl Trizol reagent (Life Technologies, Carlsbad, CA) according to manufacturer's instructions. RNA concentrations were measured at 260 nm using a NanoDrop 8000 spectrophotometer (Thermo Fisher Scientific, Wilmington, DE) and first-strand cDNA was synthesized from 1.5 μg total RNA using the high-capacity cDNA reverse transcription kit (Applied Biosystems, Foster City, CA) according to manufacturer's instructions. Specific mRNA expression was quantified by real-time PCR using SYBR Green (Applied Biosystems) and performed on an ABI 7900HT instrument (Applied Biosystems). Thermocycler conditions were as follows: 95°C for 10 min, followed by 40 PCR cycles of denaturation at 95°C for 15 s, and annealing/extension at 60°C for 1 min. Gene expression was determined using previously published primers for atrial natriuretic peptide (*ANP*), b-type natriuretic peptide *(BNP)*, Cyclooxygenase-2 *(Cox2)*, Collagen 1a1 *(Col1a1)*, Collagen 3a1 *(Col3a1)*, Galectin-3 *(Lgals3)*, Angiotensin converting enzyme *(Ace)*, ANGII type 1 receptor-*a (Agtr1a)*, and ANGII type 1 receptor-b *(Agtr1b)*. Primer sequences are listed in Supplementary Table 1. The mRNA expression levels were normalized to beta-actin and are expressed relative to the control group. Relative gene expression was determined by the ΔΔCT method. Primer specificity and purity of the final PCR product were confirmed by melting curve analysis.

### Statistical Analysis

Data were analyzed using GraphPad Prism software (version 9.0, La Jolla, CA) and are presented as individual data points and their means ± standard errors of the mean (SEM). Comparisons among different treatment groups were performed by ordinary two-way analysis of variance (ANOVA), followed by Tukey's multiple comparison *post-hoc* analysis. Comparisons between two groups were performed by unpaired student's two-tailed *t*-test. Statistical analyses for histopathologic grading were performed using the non-parametric Kruskal-Wallis test. A *p*-value of <0.05 was taken to indicate statistical significance.

## Results

### Juvenile Exposure to DOX Abrogated the Hypertrophic Response to Both ANGII and ISO

Juvenile exposure to DOX (4 mg/kg/week for 3 weeks) did not cause significant morbidity or mortality in mice, similar to our earlier study (11). In addition, treatments with either ANGII or ISO were not associated with significant morbidity or mortality, when administered to control or DOX-treated mice (Supplementary Figure 1).

Corroborating previous studies (16, 18), 2 weeks of ANGII infusion or ISO injections caused cardiac hypertrophy in control mice as demonstrated by an increase in the heart weight to tibia length (HW/TL) (Figures 1B,F). Remarkably, juvenile exposure to DOX prevented both ANGII- and ISO-induced cardiac hypertrophy as evident by a reduction in HW/TL (Figures 1B,F). To follow on this result, we measured cardiomyocyte surface area to determine if the reduction of heart weight is due to cardiomyocyte atrophy (Figures 1C,D,G,H). Mice exposed to DOX/ANGII had the smallest surface area among the groups and this group was significantly different from mice treated with ANGII only (Figures 1C,D). No statistically significant differences in cardiomyocyte surface area were observed in mice treated with ISO (Figures 1G,H). Images shown are representative images for each group, where wheat germ agglutinin staining is pseudo-colored green (Figures 1C,G).

**Figure 2:**
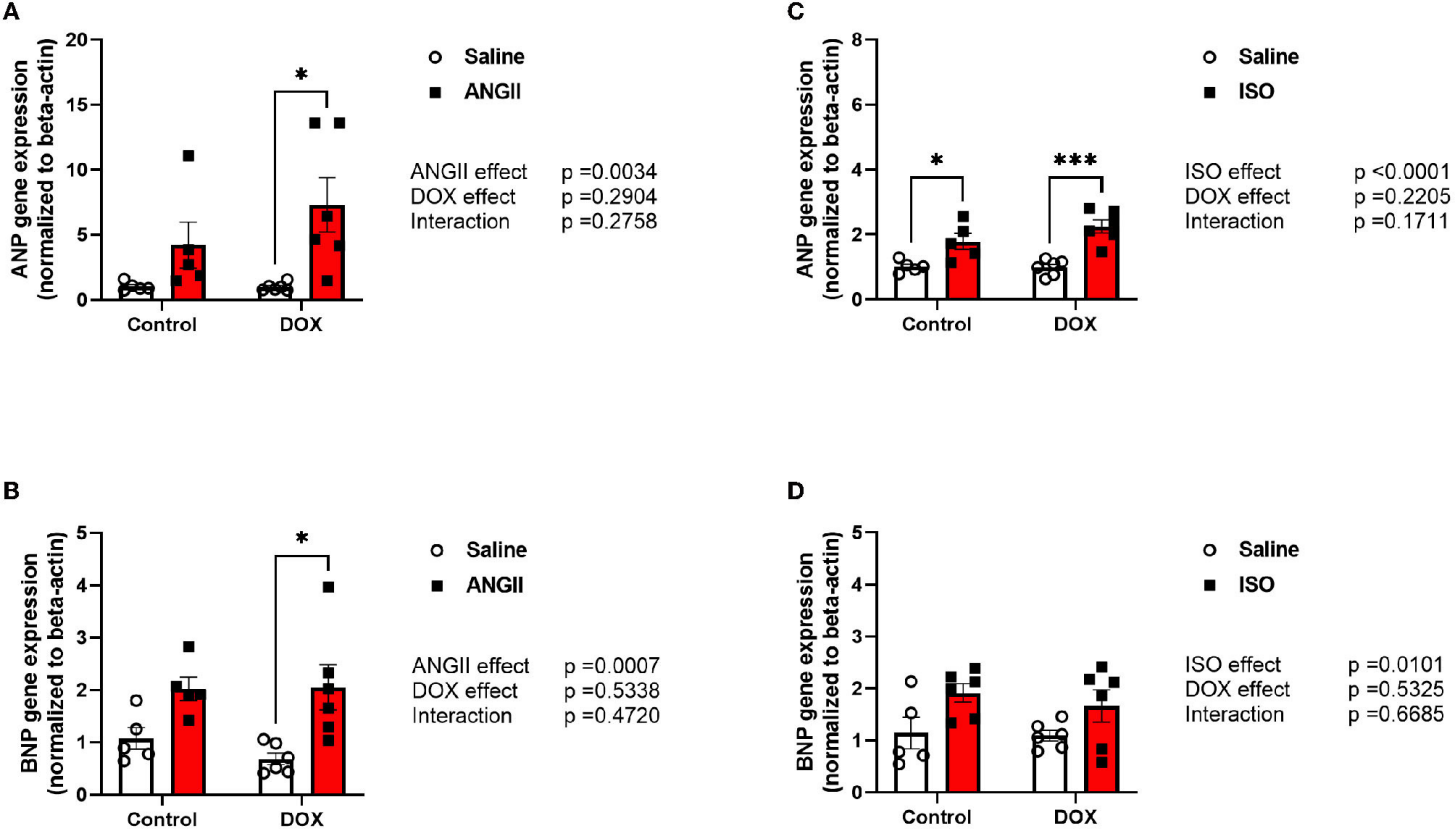
Juvenile exposure to DOX fails to correct ANGII- and ISO-induced upregulation of hypertrophic markers. Male 5-week old mice were administered DOX (4 mg/kg/week) or saline for 3 weeks and allowed to recover for 5 weeks prior to exposure to **(A,B)** ANGII (1.4 mg/kg/day for 14 days) or **(C,D)** ISO (10 mg/kg/day for 14 days). The mRNA expression of *ANP* **(A,C)** and *BNP* **(B,D)** was determined by real-time PCR (*n* = 5-6 per group); results were normalized to beta-actin and are expressed relative to the control group. Values are represented as means ± SEM. Statistical significance of pairwise comparisons was determined by two-way ANOVA with Tukey's *post-hoc* analysis (*p < 0.05, ***p < 0.001). ANGII, Angiotensin II; ANP, atrial natriuretic peptide; BNP, B-type natriuretic peptide; DOX, doxorubicin; ISO, Isoproterenol.

Measurements of ANP and BNP mRNA expressions were assessed to determine the cardiotoxicity that was induced on the heart by the pharmacological interventions. There were no differences observed in ANP and BNP mRNA expression between control and ANGII treated mice. The combination of DOX/ANGII significantly increased the expression of these markers compared to DOX alone (Figures 2A,B). On the other hand, it appears that DOX did not exacerbate the ISO mediated increases in ANP and BNP since no statistically significant differences were observed between DOX/ISO and ISO alone (Figures 2C,D).

### ANGII but Not ISO Worsens Cardiac Function in DOX-Exposed Mice

**Figure 3:**
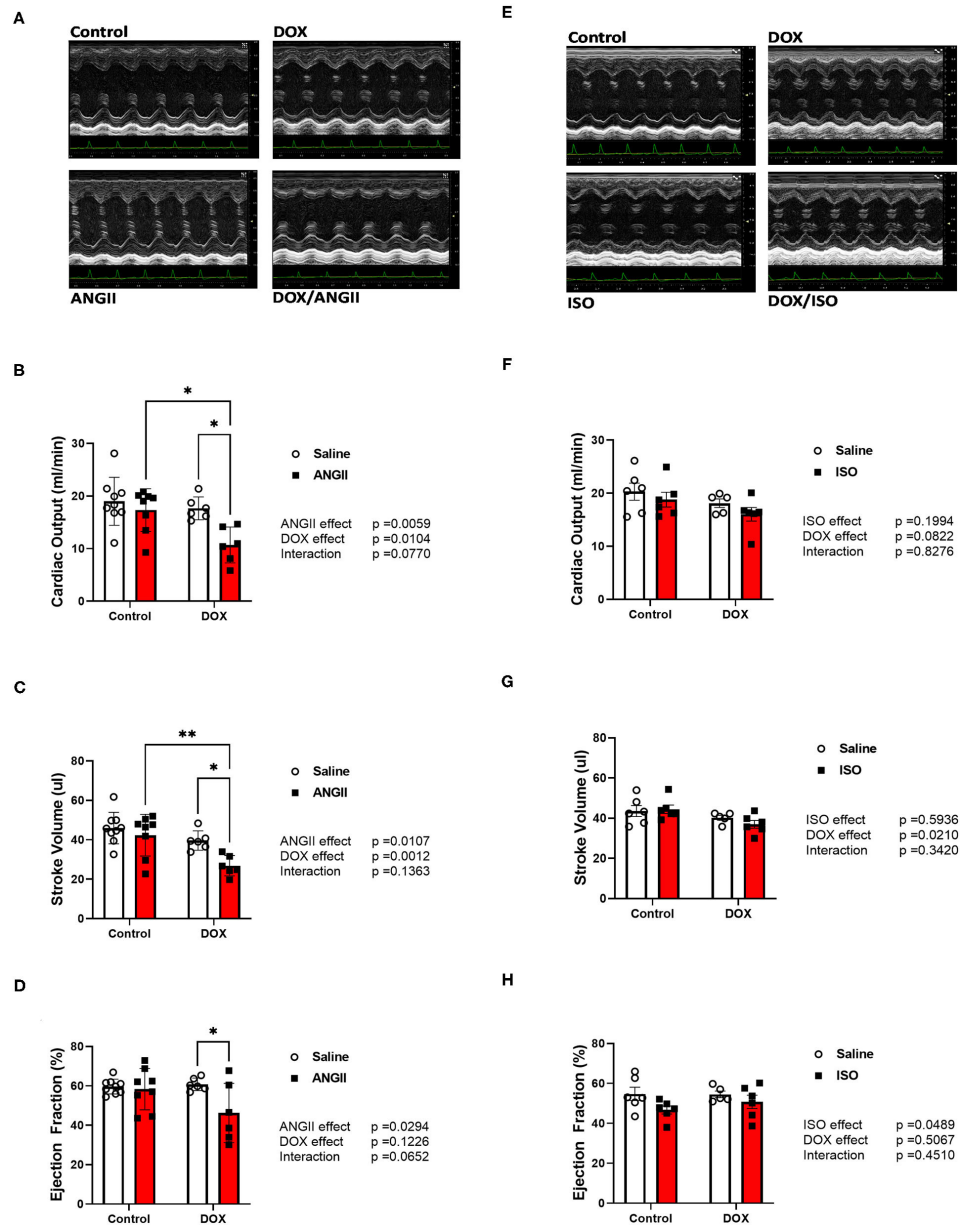
ANGII, but not ISO, worsens cardiac function in DOX-exposed mice. Male 5-week old mice were administered DOX (4 mg/kg/week) or saline for 3 weeks and allowed to recover for 5 weeks prior to exposure to **(A–D)** ANGII (1.4 mg/kg/day for 14 days) or **(E–H)** ISO (10 mg/kg/day for 14 days). Cardiac function was determined by trans-thoracic echocardiography (*n* = 5-9 per group). **(A,E)** Representative M-Mode images from parasternal short axis view of the heart. **(B,F)** Cardiac output. **(C,G)** Stroke volume. **(D,H)** Ejection fraction. Values are represented as means ± SEM. Statistical significance of pairwise comparisons was determined by two-way ANOVA with Tukey's *post-hoc* analysis (*p < 0.05, **p <0.01). ANGII, Angiotensin II; DOX, doxorubicin; ISO, isoproterenol.

There were no significant changes in systolic cardiac function 5 weeks after the last DOX treatment, as evidenced by no significant difference in ejection fraction or fractional shortening (Table 1). Cardiac output and stroke volume were significantly lower in DOX-exposed mice than that in saline-treated mice, which was associated with a reduction in LV mass and wall thickness (Table 1). Neither DOX nor ANGII alone was sufficient to significantly reduce the cardiac function in mice (Figures 3A–D); however, juvenile exposure to DOX followed by adult-onset ANGII-induced hypertension caused a significant deterioration in cardiac function parameters as shown by a decrease in cardiac output (Figure 3B), stroke volume (Figure 3C), and ejection fraction (Figure 3D). Intriguingly, when DOX-exposed mice were subjected to ISO as a second cardiovascular hit, the cardiac function of DOX/ISO-treated mice did not significantly differ from the other groups (Figures 3E–H). Tables 2, 3 show detailed echocardiography measurements after 14 days of ANGII, ISO, or saline treatment in control and DOX-treated mice.

**Table 1:** Cardiac function and morphometry measured by trans-thoracic echocardiography in control and DOX-treated mice 5 weeks following the last DOX administration.

**Parameter**	**Control**	**DOX**
	**mean (SEM)**	**mean (SEM)**
CO (ml/min)	18.19 (0.6730)	15.59^**^ (0.4575)
SV (μl)	42.73 (1.608)	37.51^**^ (0.6720)
EF (%)	54.72 (1.792)	55.18 (1.879)
FS (%)	28.33 (1.121)	28.53 (1.162)
LV Mass (mg)	128.0 (4.310)	101.4^****^ (2.111)
LVESV (μl)	37.95 (2.564)	32.90 (2.742)
LVEDV (μl)	84.02 (3.644)	75.60 (2.650)
LVAW;s (mm)	1.373 (0.02731)	1.280 (0.04129)
LVAW;d (mm)	1.017 (0.02067)	0.9286^*^ (0.02676)
LVPW;s (mm)	1.124 (0.03176)	0.9752^***^ (0.02614)
LVPW;d (mm)	0.7967 (0.02410)	0.6684^****^ (0.01267)
HR (bpm)	427 (5)	416 (11)

*Values are presented as mean ± standard error of the mean (SEM) (N = 22-23). Statistical significance was determined using an unpaired t-test*.

* *p < 0.05*,

** *p < 0.01*,

*** *p < 0.001*,

**** *p < 0.0001 vs. control*.

*DOX, doxorubicin; CO, cardiac output; SV, stroke volume; EF, ejection fraction; FS, fractional shortening; LV, left ventricle; LVESV, LV end systolic volume; LVEDV, LV end diastolic volume; LVAW;s, LV anterior wall systole; LVAW;d, LV anterior wall diastole; LVPW;s, LV posterior wall systole; LVPW;d, LV posterior wall diastole; HR, heart rate*.

**Table 2:** Cardiac function and morphometry measured by trans-thoracic echocardiography in control, ANGII, DOX, DOX/ANGII-treated mice.

**Parameter**	**Control** **mean (SEM)**	**ANGII** **mean (SEM)**	**DOX** **mean (SEM)**	**DOX/ANGII** **mean (SEM)**	**ANGII effect**		**DOX effect**		**Interaction effect**	
					**Effect size (%)**	***P*-value**	**Effect size (%)**	***P*-value**	**Effect size (%)**	***P*-value**
CO (ml/min)	18.998 (1.518)	17.328 (1.434)	17.667 (0.885)	10.695^b^, ^c^ (1.393)	20.67	0.0059	17.55	0.0104	7.776	0.0770
SV (μl)	45.953 (2.659)	42.330 (3.744)	39.634 (2.012)	26.866^b, c^ (2.117)	16.02	0.0107	28.29	0.0012	4.986	0.1363
EF (%)	59.651 (1.258)	58.340 (3.707)	60.786 (1.299)	46.235^c^ (6.151)	15.10	0.0294	7.222	0.1226	10.52	0.0652
FS (%)	31.432 (0.844)	32.044 (0.843)	30.825 (2.498)	23.195 (3.701)	7.473	0.1198	13.57	0.0397	10.31	0.0701
LV mass (mg)	129.918 (6.306)	147.820 (6.334)	110.631 (7.536)	119.872 (9.024)	8.849	0.0733	26.80	0.0033	0.9011	0.5562
LVESV (μl)	31.627 (2.931)	31.681 (5.289)	26.048 (2.645)	34.595 (6.427)	3.360	0.3536	0.3225	0.7720	3.276	0.3595
LVEDV (μl)	77.578 (5.390)	74.009 (7.285)	65.682 (4.635)	61.460 (6.247)	1.323	0.5393	13.03	0.0621	0.009296	0.9588
LVAW;s (mm)	1.475 (0.055)	1.614 (0.083)	1.412 (0.067)	1.340^b^ (0.029)	0.7565	0.6144	18.94	0.0172	7.446	0.1220
LVAW;d (mm)	1.087 (0.030)	1.166 (0.038)	1.094 (0.053)	1.128 (0.020)	7.913	0.1558	0.5889	0.6929	1.237	0.5677
LVPW;s (mm)	1.125 (0.041)	1.310^a^ (0.060)	1.035 (0.014)	1.097^b^ (0.055)	14.74	0.0183*	22.06	0.0049	3.690	0.2184
LVPW;d (mm)	0.792 (0.025)	0.936^a^ (0.046)	0.701 (0.019)	0.898^c^ (0.052)	42.00	0.0002	5.985	0.1046	1.026	0.4920
HR (bpm)	411 (16)	413 (13)	446 (8)	390 (26)	8.487	0.1244	0.5206	0.6971	9.698	0.1016

*Values are presented as mean ± standard error of the mean (SEM) (N = 6-9). Statistical significance was determined using Two-way ANOVA with Tukey post-hoc test*.

a *significant difference (p < 0.05) vs. control*,

b *significant difference (p < 0.05) vs. ANGII*,

c *significant difference (p < 0.05) vs. DOX*.

*DOX, doxorubicin; ANGII, angiotensin II; CO, cardiac output; SV, stroke volume; EF, ejection fraction; FS, fractional shortening; LV, left ventricle; LVESV, LV end systolic volume; LVEDV, LV end diastolic volume; LVAW;s, LV anterior wall systole; LVAW;d, LV anterior wall diastole; LVPW;s, LV posterior wall systole; LVPW;d, LV posterior wall diastole; HR, heart rate*.

**Table 3:** Cardiac function and morphometry measured by trans-thoracic echocardiography in control, ISO, DOX, and DOX/ISO-treated mice.

**Parameter**	**Control** **mean (SEM)**	**ISO** **mean (SEM)**	**DOX** **mean (SEM)**	**DOX/ISO** **mean (SEM)**	**ISO effect**		**DOX effect**		**Interaction effect**	
					**Effect size (%)**	***P*-value**	**Effect size (%)**	***P*-value**	**Effect size (%)**	***P*-value**
CO (ml/min)	20.277 (1.611)	18.780 (1.382)	18.099 (0.816)	16.006 (1.281)	7.235	0.1994	13.78	0.0822	0.1995	0.8276
SV (μl)	43.548 (2.741)	44.475 (2.125)	40.238 (1.203)	36.981 (1.920)	1.095	0.5936	23.52	0.0210*	3.530	0.3420
EF (%)	54.733 (3.368)	46.709 (1.998)	54.491 (1.682)	50.739 (3.336)	18.03	0.0489	1.865	0.5067	2.372	0.4547
FS (%)	28.337 (2.205)	23.328 (1.151)	27.932 (1.088)	25.679 (2.004)	17.80	0.0510	1.277	0.5834	2.563	0.4390
LV Mass (mg)	123.532 (4.444)	142.947^a^ (4.850)	111.599 (5.807)	115.680^b^ (4.893)	13.08	0.0291	36.40	0.0009	5.570	0.1400
LVESV (μl)	38.702 (4.164)	52.299 (4.359)	34.158 (2.724)	38.550 (4.384)	16.04	0.0404	16.59	0.0375	4.200	0.2744
LVEDV (μl)	80.931 (4.542)	100.300^a^ (5.185)	81.370 (2.362)	79.407^b^ (4.553)	11.08	0.0659	15.30	0.0334	16.64	0.0273
LVAW;s (mm)	1.330 (0.087)	1.307 (0.062)	1.313 (0.074)	1.242 (0.062)	2.127	0.5230	1.685	0.5693	0.5551	0.7432
LVAW;d (mm)	1.025 (0.046)	1.076 (0.025)	0.983 (0.047)	1.010 (0.054)	3.619	0.3911	6.931	0.2395	0.3312	0.7935
LVPW;s (mm)	1.114 (0.054)	0.989 (0.026)	0.959 (0.070)	1.047 (0.058)	0.4790	0.7384	3.546	0.3681	16.95	0.0582
LVPW;d (mm)	0.794 (0.042)	0.780 (0.015)	0.690 (0.033)	0.734 (0.028)	0.9204	0.6328	22.68	0.0262	3.506	0.3552
HR (bpm)	463 (10)	421 (14)	449 (11)	430 (18)	19.78	0.0390	0.1030	0.8746	2.785	0.4158

*Values are presented as mean ± standard error of the mean (SEM) (N = 5-6). Statistical significance was determined using Two-way ANOVA with Tukey post-hoc test*.

a *Significant difference (p < 0.05) vs. control*,

b *significant difference (p < 0.05) vs. ISO*.

*DOX, doxorubicin; ISO, isoproterenol; CO, cardiac output; SV, stroke volume; EF, ejection fraction; FS, fractional shortening; LV, left ventricle; LVESV, LV end systolic volume; LVEDV, LV end diastolic volume; LVAW;s, LV anterior wall systole; LVAW;d, LV anterior wall diastole; LVPW;s, LV posterior wall systole; LVPW;d, LV posterior wall diastole; HR, heart rate*.

### ANGII but Not ISO Worsens Cardiac Fibrosis in DOX-Exposed Mice

**Figure 4:**
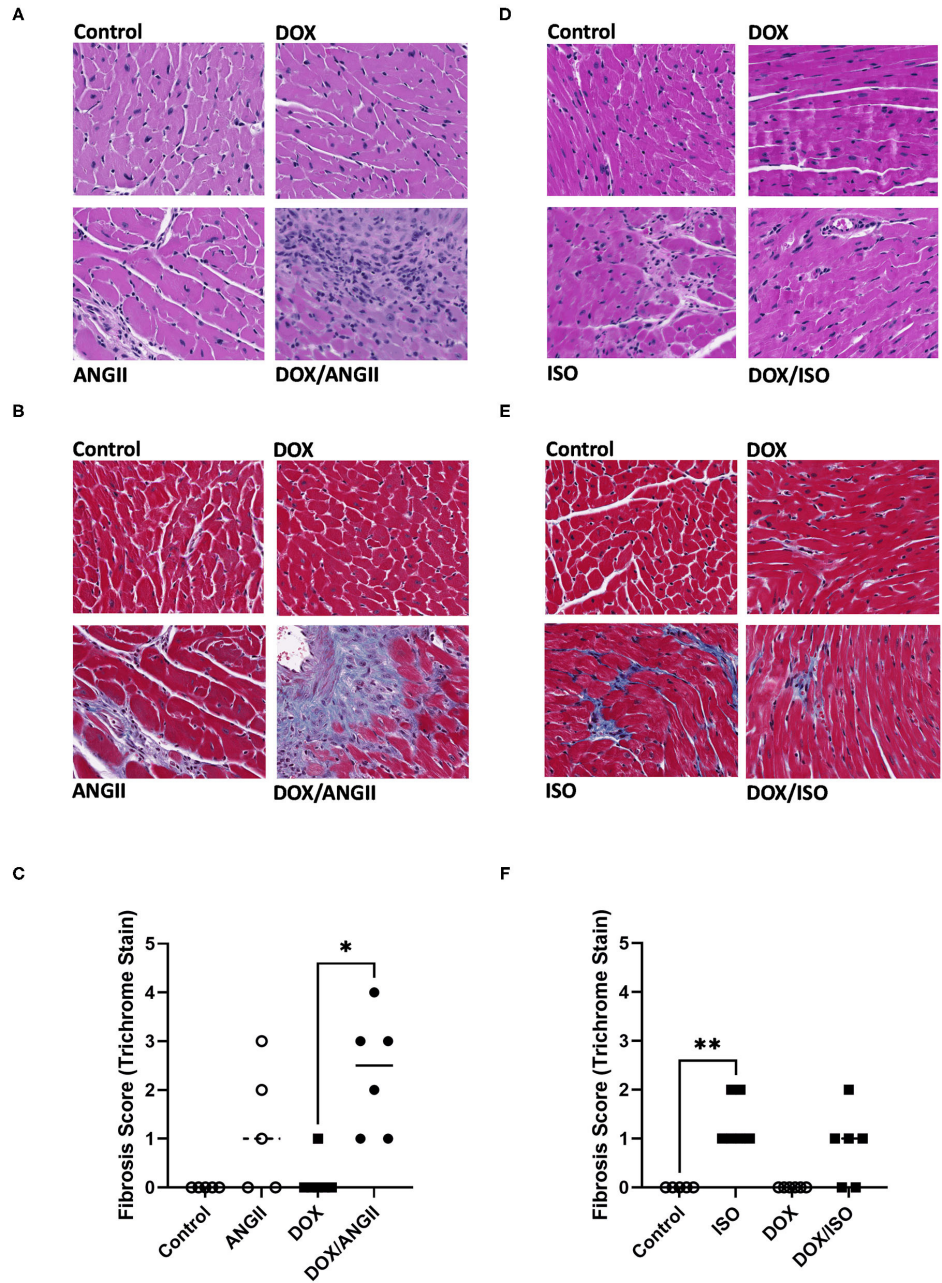
ANGII, but not ISO, worsens cardiac fibrosis in DOX-exposed mice. Male 5-week old mice were administered DOX (4 mg/kg/week) or saline for 3 weeks and allowed to recover for 5 weeks prior to exposure to **(A–C)** ANGII (1.4 mg/kg/day for 14 days) or **(D–F)** ISO (10 mg/kg/day for 14 days). Representative images from H&E **(A,D)** and Masson's trichrome stained heart sections **(B,E)**. **(C,F)** Semi-quantification of fibrosis score derived from Masson's trichrome stain (*n* = 5-6 per group). Statistical significance was determined by non-parametric Kruskal-Wallis test (*p < 0.05, **p < 0.01). ANGII, Angiotensin II; DOX, doxorubicin; H&E, hematoxylin and eosin; ISO, isoproterenol.

**Figure 5:**
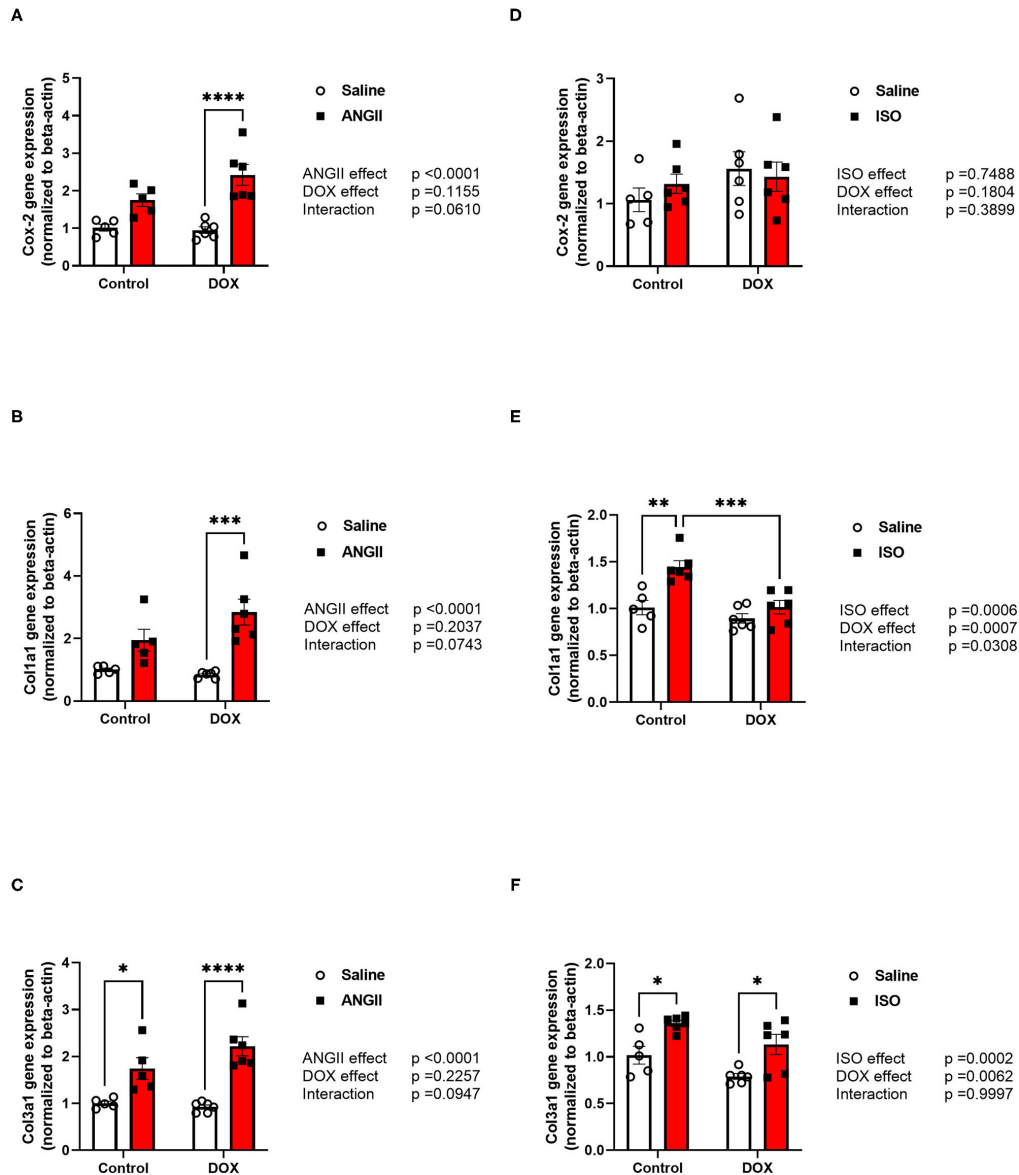
ANGII, but not ISO, exacerbates the upregulation of inflammatory and fibrotic markers in DOX-exposed mice. Male 5-week old mice were administered DOX (4 mg/kg/week) or saline for 3 weeks and allowed to recover for 5 weeks prior to exposure to **(A–C)** ANGII (1.4 mg/kg/day for 14 days) or **(D–F)** ISO (10 mg/kg/day for 14 days). The mRNA expression of **(A,D)** the inflammatory marker *Cox-2*, and the fibrotic markers **(B,E)** *Col1a1* and **(C,F)** *Col3a1* was determined by real-time PCR (*n* = 5-6 per group). Results were normalized to beta-actin and are expressed relative to the control group. Statistical significance of pairwise comparisons was determined by two-way ANOVA with Tukey's *post-hoc* analysis (^*^p < 0.05, **p < 0.01, ***p < 0.001, ****p < 0.0001). ANGII, Angiotensin II; DOX, doxorubicin; ISO, isoproterenol.

Histopathology analysis using H&E and Masson's trichrome stains revealed marked inflammatory cell infiltration and cardiac fibrosis in the DOX/ANGII group as compared to the control (Figures 4A–C). Although a few mice in the DOX and ANGII groups showed signs of cardiac fibrosis at varying degrees, neither DOX nor ANGII alone was sufficient to cause a statistically significant effect on cardiac fibrosis (Figure 4C). The combination of DOX and ANGII significantly increased fibrosis suggesting that DOX potentiates the fibrosis inducing action of ANGII. On the other hand, ISO treatment caused modest, but statistically significant, cardiac fibrosis which was not exacerbated by DOX treatment (Figures 4D–F). To ascertain the molecular determinants of the observed fibrotic changes, we measured gene expression of several inflammatory and fibrotic markers. ANGII caused a significant induction of the inflammatory marker *Cox-2* (Figure 5A) and the fibrotic markers, *Col1a1* and *Col3a1* (Figures 5B,C). Juvenile exposure to DOX mildly but not significantly exacerbated ANGII-induced upregulation of inflammatory and fibrotic markers (Figures 5A–C). Marked increases in inflammatory and fibrotic markers were observed in DOX/ANGII treated mice compared to mice only treated with DOX. On the other hand, DOX/ISO had no exacerbating effect on *Cox-2* or *Col3a1* expression (Figures 5D,F), while there was a significant reduction in *Col1a1* expression in the DOX/ISO treated mice compared to ISO alone (Figure 5E).

**Figure 6:**
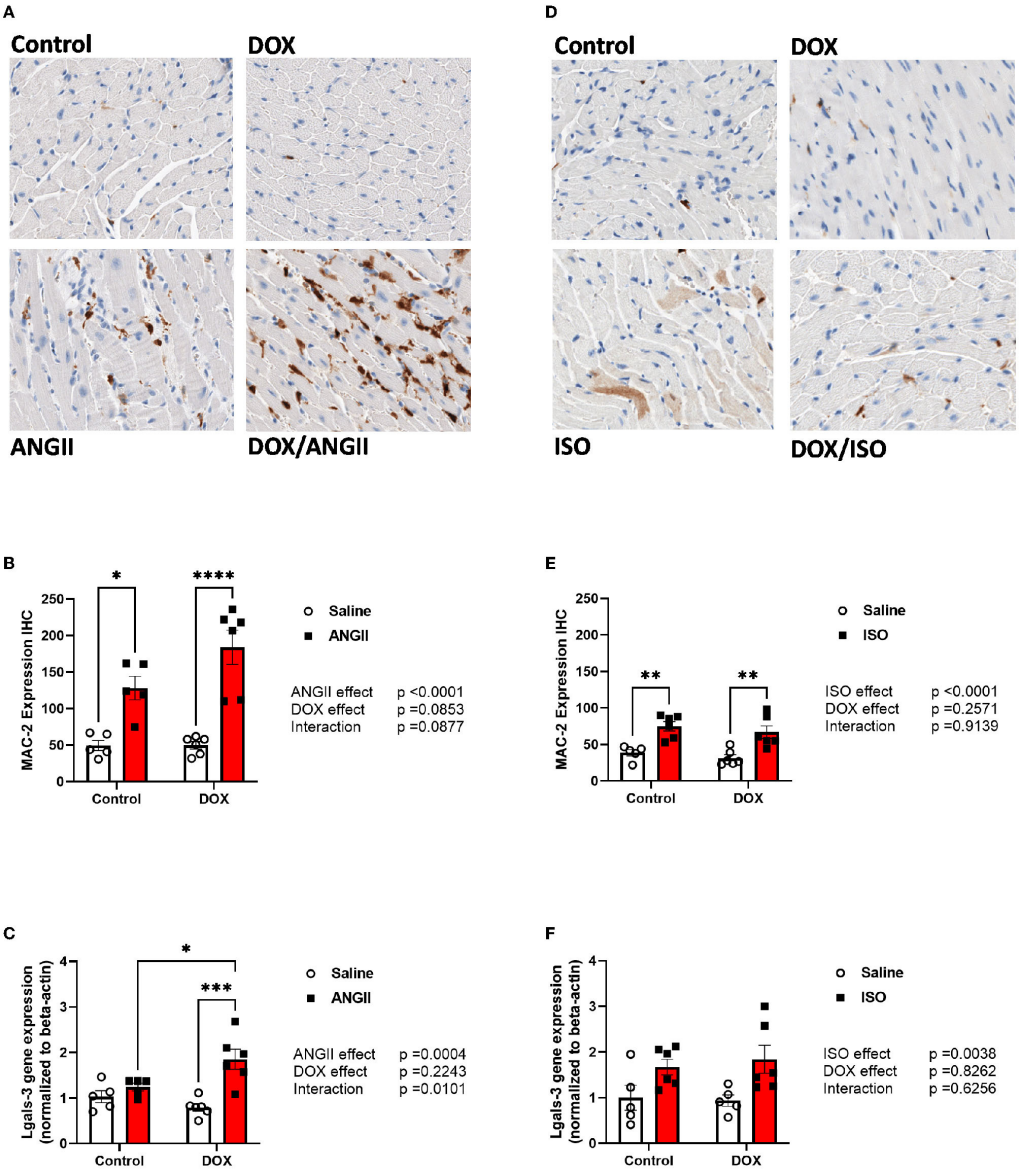
ANGII, but not ISO, exacerbates the upregulation of MAC-2 in DOX-exposed mice. Male 5-week old mice were administered DOX (4 mg/kg/week) or saline for 3 weeks and allowed to recover for 5 weeks prior to exposure to **(A–C)** ANGII (1.4 mg/kg/day for 14 days) or **(D–F)** ISO (10 mg/kg/day for 14 days). Representative images from **(A,D)** MAC-2 stained heart sections. **(B,E)** Semi-quantification of MAC-2 positive cells (*n* = 5-6 per group). The mRNA expression of **(C,F)** *Lgals-3* was determined by real-time PCR (*n* = 5-6 per group). Results were normalized to beta-actin and are expressed relative to the control group. Statistical significance of pairwise comparisons was determined by two-way ANOVA with Tukey's *post-hoc* analysis (*p < 0.05, **p < 0.01, ***p < 0.001, ****p < 0.0001). ANGII, Angiotensin II; DOX, doxorubicin; ISO, isoproterenol; Lgals-3, lectin, galactoside-binding, soluble-3.

Since macrophage infiltration plays an important role in cardiac fibrosis, we measured the expression the fibrotic marker galectin-3 by measuring MAC-2 positive cells by immunohistochemistry as well as the gene expression of galectin-3 *(Lgals-3)*. As expected, ANGII caused a significant increase in the number of MAC-2 positive cells (Figures 6A,B) but not a significant induction of *Lgals-3* gene expression (Figure 6C). In agreement with the exacerbation of cardiac fibrosis in the DOX/ANGII group, DOX/ANGII-treated mice had the highest number of MAC-2 positive cells (Figures 6A,B). Juvenile exposure to DOX significantly aggravated ANGII-induced upregulation of *Lgals-3* gene expression (Figure 6C). ISO caused a modest but significant increase in the number of MAC-2 positive cells (Figures 6D,E) and caused a significant upregulation of *Lgal-3* gene expression (Figure 6F). However, juvenile exposure to DOX did not change the effects of ISO on these parameters (Figures 6D–F).

### Perturbed RAAS Gene Expression in the DOX/ANGII Model

**Figure 7:**
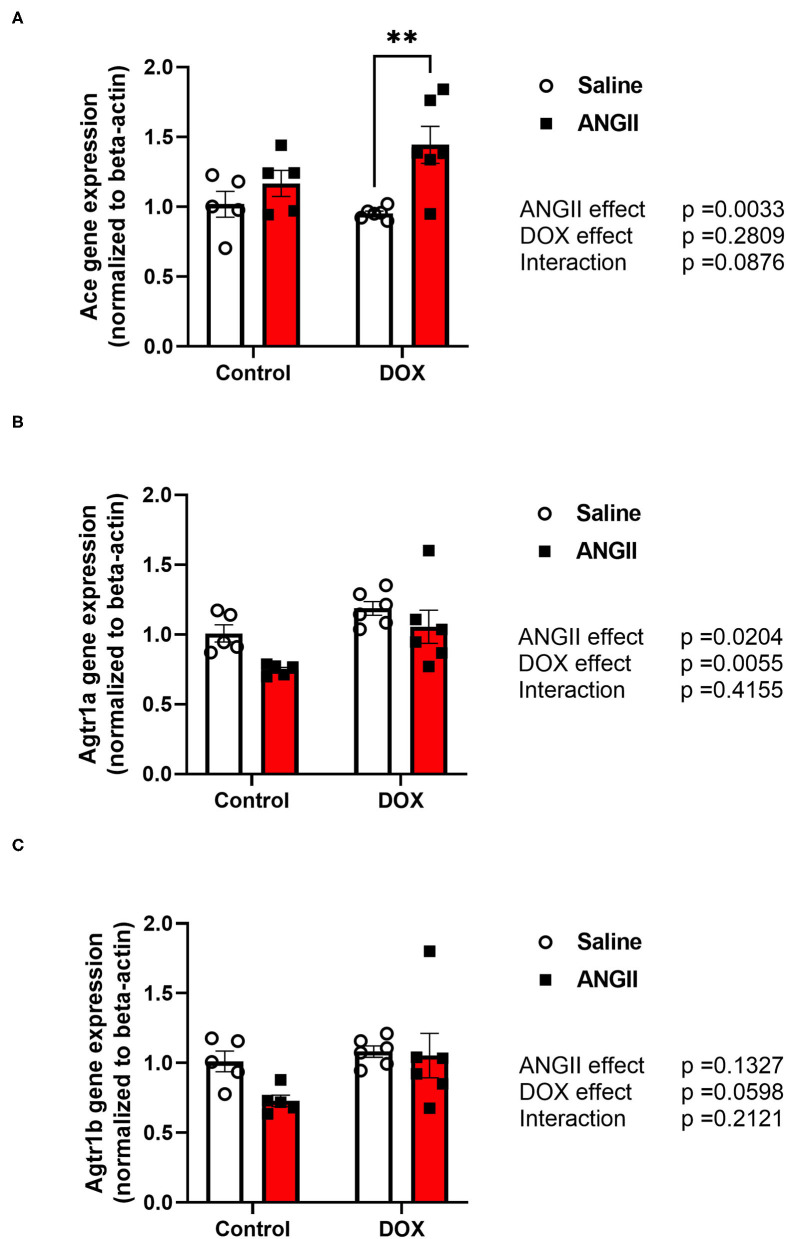
Perturbation of the RAAS pathway in the DOX/ANGII model. Male 5-week old mice were administered DOX (4 mg/kg/week) or saline for 3 weeks and allowed to recover for 5 weeks prior to exposure to ANGII (1.4 mg/kg/day for 14 days). The mRNA expression of **(A)** *Ace*, **(B)** *Agtr1a*, and **(C)** *Agtr1b* was determined by real-time PCR (*n* = 5-6 per group). Results were normalized to beta-actin and are expressed relative to the control group. Statistical significance of pairwise comparisons was determined by two-way ANOVA with Tukey's *post-hoc* analysis (**p < 0.01). Ace, Angiotensin converting enzyme; Agtr1a, Angiotensin II type1 receptor-a; Agtr1b, Angiotensin II type1 receptor-b; ANGII, Angiotensin II; DOX, doxorubicin.

Since DOX has been shown to alter the renin-angiotensin-aldosterone-system (RAAS) in different ways (19), we sought to determine whether DOX-induced alteration in the RAAS may have played a role in the detrimental synergy between DOX and ANGII. To this end, we determined the effects of DOX, ANGII, and DOX/ANGII on expression of the RAAS genes in the heart. Interestingly, the gene expression of angiotensin converting enzyme (*Ace*) was significantly upregulated in the DOX/ANGII group compared to DOX alone (Figure 7A). Next, measurements of the *Atgr1a* and *Atgr1b*, the gene encoding for the ANGII type 1 receptor (AT1) were measured and no statistically significant differences were observed among the groups (Figures 7B,C).

## Discussion

**Figure 8:**
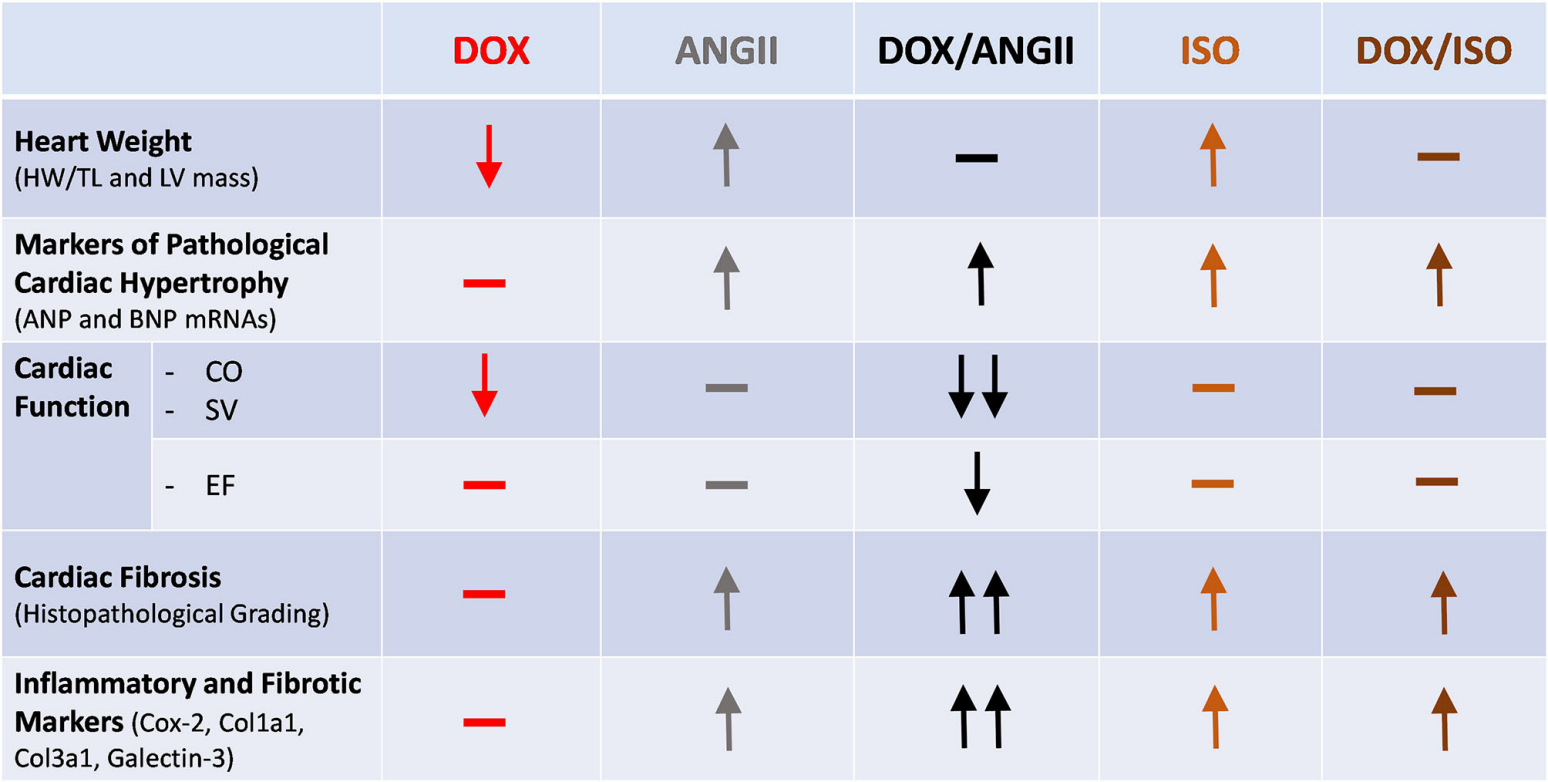
Divergent cardiac effects of ANGII and ISO in adult mice pre-exposed to DOX as juveniles. Male 5-week old mice were administered DOX (4 mg/kg/week) or saline for 3 weeks and allowed to recover for 5 weeks prior to exposure to ANGII (1.4 mg/kg/day for 14 days) or ISO (10 mg/kg/day for 14 days). Juvenile exposure to DOX prevented both ANGII- and ISO-induced cardiac hypertrophy, but failed to correct the upregulation in hypertrophic markers. ANGII, but not ISO, worsened cardiac function, exacerbated cardiac fibrosis, and upregulated inflammatory and fibrotic markers in DOX-exposed mice. ANGII, Angiotensin II; DOX, doxorubicin; ISO, isoproterenol.

Childhood cancer survivors have a considerably increased risk for premature cardiovascular diseases (20), with an estimated 15 times higher risk of heart failure than their siblings who did not have cancer (2). Nearly 50% of pediatric cancer patients receive anthracyclines such as doxorubicin (DOX), which are known to cause cardiotoxicity (21). Although the risk of anthracycline-induced cardiotoxicity increases with a higher anthracycline cumulative dose (22), latent (subclinical) cardiotoxicity occurs in children who receive low doses of anthracyclines (5–7). Anthracycline-induced subclinical cardiotoxicity is characterized by reduction in the left ventricular mass, mild cardiac fibrosis, and modest decline in ejection fraction (5–7). This latent cardiotoxicity can be unmasked and overt cardiomyopathy precipitated by other cardiovascular risk factors in adulthood, in a two-hit manner (10). We designed the current experimental protocol to mimic the scenario in cancer survivors that undergo DOX treatment at young age. With this protocol, we are able to show that latent cardiotoxicity caused by juvenile exposure to DOX is exacerbated when adult mice undergo a hypertensive “second-hit” on the heart. We used ANGII and ISO as two pharmacological agents that both increase the stress on the heart through distinct mechanisms. In this report, we show that the combination of DOX and ANGII causes the most changes to heart size, cardiac function and fibrosis. While the combination of DOX and ISO shows modest changes in heart size, cardiac function and fibrosis are not affected (Figure 8).

Most preclinical models of juvenile DOX-induced cardiotoxicity used high cumulative doses of DOX that were enough to cause immediate or delayed cardiac dysfunction (23–26). Although clinically relevant, animal models for juvenile DOX cardiotoxicity have rarely adopted the two-hit models. Huang *et al*. demonstrated that low doses of DOX administered to very young mice, at postnatal day 5, did not cause immediate cardiac dysfunction. DOX-exposed mice developed normally and had no obvious cardiac dysfunction as adults. However, juvenile exposure to DOX exacerbated cardiac pathology in response to an adult-onset pathologic stimulus (myocardial infarction) and even a physiologic stimulus (swimming exercise) (27). Since hypertension is the most significant cardiovascular risk factor for all adverse cardiac events, including heart failure and cardiac death, in anthracycline-treated childhood cancer survivors (10), we have recently developed another two-hit mouse model of juvenile DOX-induced latent cardiotoxicity that is exacerbated by adult-onset ANGII-induced hypertension (11). Similar to Huang et al., we demonstrated that low doses of DOX (4 mg/kg/week for 3 weeks) did not cause immediate cardiac dysfunction in juvenile mice, but predisposed to late-occurring detrimental cardiovascular changes when the mice were challenged by ANGII-induced hypertension (11). However, unlike Huang et al., our DOX administration regimen starts at 5 weeks of age, equivalent to 10 years in human life. Therefore, these dosage regimens model latent cardiotoxicity in anthracycline-treated pediatric cancer patients who do not immediately develop overt cardiac dysfunction but are left with “weaker” hearts that predispose them to other cardiovascular insults, corroborating the findings of several clinical studies (5, 28–31).

Nevertheless, it is still not known how this low-dose DOX regimen would predispose the heart to other cardiovascular pathologic stimuli that do not cause hypertension. To answer this question, we subjected control and DOX-treated mice to a regimen of ISO injections (10 mg/kg/day for 14 days). ISO is a non-specific beta-adrenoceptor agonist that is commonly used to induce a dose-dependent cardiac pathology without elevating blood pressure (12–15). We have previously demonstrated that this dosage regimen causes cardiac hypertrophy, mild cardiac dysfunction, and modest cardiac fibrosis in C57BL/6N male mice (12). In the current study, we characterize the DOX/ANGII model in parallel to the DOX/ISO model to better understand why hypertension is the most significant risk factor for cardiovascular morbidity and mortality in anthracycline-treated childhood cancer survivors.

Cardiac atrophy and thinning of the LV ventricular walls are common late effects of anthracycline therapy in childhood cancer survivors (32–35). Intriguingly, a study has shown that reduction in the LV mass is associated with worsening of heart failure symptomatology independent of LV ejection fraction in adult cancer survivors (36), demonstrating the predictive value of LV mass. However, the association between LV mass and heart failure symptomatology has not been determined in childhood cancer survivors. We previously demonstrated that juvenile exposure to DOX prevented the adaptive cardiac hypertrophy in response to ANGII-induced hypertension (11). However, it is not known whether juvenile exposure to DOX would also prevent adaptive cardiac hypertrophy in response to other hypertrophic stimuli. In the current study, juvenile exposure to DOX prevented the adaptive cardiac hypertrophy in response to both ANGII and ISO. Indeed, ANGII and ISO cause cardiac hypertrophy *via* different pathways. ANGII induces cardiac hypertrophy directly through activating the AT1 receptors on cardiomyocytes and indirectly through elevating the afterload (37). In contrast, ISO activates the beta-adrenoceptors on cardiomyocytes to elicit a direct hypertrophic effect (37). ISO-induced tachycardia may also contribute to its hypertrophic effect indirectly. The ability of DOX to prevent cardiac hypertrophy in response to both pathologic stimuli suggest that DOX interferes with common downstream pathways fundamental to the development of cardiac hypertrophy.

Although high-dose DOX causes cardiac atrophy due to apoptotic and necrotic cell death and loss of cardiomyocytes (38), experimental studies using low/divided-dose DOX have suggested that DOX-induced cardiomyocyte atrophy is the main culprit leading to cardiac atrophy and reduction of LV mass with minimal apoptotic cell death (39, 40). We demonstrate that mice with juvenile exposure to DOX had the smallest cardiomyocyte surface area after ANGII exposure. These experimental observations have recently been supported by a clinical study reporting that the reduction in LV mass after anthracycline therapy is due to cardiomyocyte atrophy in breast cancer patients (41). Another recent preclinical study has shown that acute DOX administration causes dose-dependent cardiac atrophy that parallels the decrease in contractile function (39). In our current study, we demonstrate that chronic administration of low-dose DOX caused cardiac atrophy without reducing the contractile function of the heart. The contractile function of the heart was only affected when the juvenile exposure of DOX was followed by ANGII-induced hypertension. ANGII cause pathologic cardiac hypertrophy characterized by the induction of fetal gene expression such as *ANP* and *BNP*, adverse cardiac remodeling, and reduction in cardiac function parameters. Therefore, in the current work, we determined the effect of juvenile DOX exposure on these parameters. Although we previously showed that juvenile exposure to DOX induced *ANP* gene expression 1 week after the last DOX injection (11), there is no significant change in *ANP* or *BNP* in DOX-exposed mice 7 weeks after the last DOX injection. Only the combination of DOX/ANGII was able to significantly elevate the markers of pathological hypertrophy. Although juvenile exposure to DOX prevented the hypertrophic growth of the heart in response to ANGII, it did not abrogate the molecular determinants of pathological cardiac hypertrophy induced by these stimuli.

We also determined the effect of juvenile exposure to DOX on cardiac remodeling in response to both ANGII and ISO. In our previous study describing the DOX/ANGII model (11), the effect on cardiac fibrosis had not been determined. In the current study, we demonstrate that juvenile exposure to DOX did not cause significant cardiac fibrosis in naïve mice, but it exacerbated ANGII-induced cardiac fibrosis. The exacerbated cardiac fibrosis was associated with a marked upregulation in several inflammatory and fibrotic markers in the DOX/ANGII-treated mice. Importantly, MAC-2 positive cells and the expression of *Lgals-3* gene encoding galectin-3 were much higher in hearts of DOX/ANGII-treated mice than in hearts of mice receiving either DOX or ANGII alone. In contrast to cardiac atrophy, which is a consistent feature of anthracycline-induced cardiotoxicity, the prevalence and extent of cardiac fibrosis in anthracycline-treated childhood cancer survivors is controversial. In a cohort of childhood cancer survivors, the prevalence of left ventricular and right ventricular fibrosis was 9 and 38%, respectively; however, these values were not compared to a healthy control group (42). Some studies report anthracycline-treated childhood cancer survivors to show modest myocardial fibrosis as evident by an increased extracellular volume fraction (35). On the other hand, other studies demonstrate the absence of a statistically significant increase in myocardial fibrosis in survivors compared to healthy control subjects (43, 44). Since there is no clinical data reporting the association between myocardial fibrosis and cardiovascular risk factors in anthracycline-treated survivors, it may be possible that the discrepancy in these clinical studies arise from the confounding effect of other cardiovascular diseases, particularly hypertension.

In contrast to the DOX/ANGII model, juvenile exposure to DOX did not exacerbate ISO-induced cardiac fibrosis. Surprisingly, the gene expression of the fibrotic marker collagen 1a1 was lower in DOX/ISO-treated mice as compared to mice treated with ISO alone. DOX-induced cardiotoxicity has been shown to attenuate the acute effects of ISO on the heart including its positive inotropic effect (45), acute decrease of myocardial stiffness (46), and stimulation of adenylyl cyclase (47). Nevertheless, the impact of DOX exposure on the chronic effects of ISO has not been previously reported. ANGII-induced increase in afterload coupled with DOX-induced thinning of the left ventricular walls is expected to markedly increase ventricular wall stress according to the Law of LaPlace. Since ISO does not increase the afterload, its effects on the heart of DOX-exposed mice would be expected to be much milder.

DOX-induced cardiotoxicity has been shown to be more severe in hypertensive experimental animals than in normotensive ones (48–50). An important distinction between these studies and our model is the fact that these studies administered DOX to already hypertensive animals, while in our model DOX is administered to young normotensive mice then challenged by ANGII-induced hypertension in their adult life, 5 weeks after the last DOX injection. In an attempt to determine the mechanism of the detrimental synergy between juvenile exposure to DOX and adult-onset ANGII-induced hypertension, we determined the effect of these experimental conditions on the renin-angiotensin-aldosterone-system (RAAS) genes. In the current study, juvenile exposure to DOX had no significant effect on the expression of *Ace, Agtr1a*, and *Agtr1b* genes. Nevertheless, there was a significant upregulation in *Ace* gene expression in the DOX/ANGII group. Similarly, juvenile exposure to DOX prevented ANGII-induced downregulation of *Agtr1a*, which encodes the AT1 receptor. ANGII mediates its detrimental effects *via* the AT1 receptors, while AT2 receptors mediate cardioprotective effects. DOX has been previously shown to alter the RAAS in different ways (19). DOX has been shown to significantly increase the expression of AT1 receptors and reduce that of AT2 receptors in a rat model of DOX-induced heart failure (51). Although there was no significant change in the mRNA expression of RAAS genes in the hearts of rabbits treated with a single dose of DOX (52), the plasma and myocardial levels of ANGII were increased three-fold in a rat model of DOX-induced heart failure (53). DOX treatment has also been shown to increase myocardial ACE activity in the cardiac tissues of hamsters (54). Intriguingly, angiotensin receptor blockers (ARBs) have been shown to ameliorate anthracycline-induced cardiotoxicity in animal models (55–57). Importantly, a recent meta-analysis shows that RAAS antagonists were the most efficient drugs to prevent anthracycline-induced cardiotoxicity with 84% risk reduction (58).

The current study has some limitations that warrant discussion. First, we have not measured the blood pressure in our experimental groups. We previously reported that juvenile exposure to DOX caused an increase in blood pressure, which was further exacerbated by ANGII infusion (11). ISO is a beta-adrenergic agonist that does not increase blood pressure, as previously reported by several other investigators (14). We also did not measure the plasma levels of natriuretic peptides, ANP and BNP. Although the induction of fetal gene expression as a hallmark of pathologic hypertrophy is usually assessed by measuring the gene expression of ANP and BNP (59, 60), measuring plasma levels of these peptides would have strengthened our conclusions.

In conclusion, this study shows that juvenile exposure to DOX differentially exacerbates ANGII—but not ISO-induced adverse cardiac remodeling. There was a marked detrimental synergy between juvenile exposure to DOX followed by ANGII-induced hypertension, which resulted in cardiac dysfunction and adverse cardiac remodeling. This preclinical mouse model highlights the clinical finding that hypertension is the most significant risk factor for heart failure in anthracycline-treated childhood cancer survivors. Since ANGII may cause cardiac damage through direct mechanism beyond elevating blood pressure, future studies are planned to delineate the mechanisms of these deleterious effects by targeting elements of RAAS system.

## Data Availability Statement

The raw data supporting the conclusions of this article will be made available by the authors, without undue reservation.

## Ethics Statement

The animal study was reviewed and approved by Institutional Animal Care and Use Committee (IACUC) at the University of Minnesota (Protocol ID:1807-36187A).

## Author Contributions

MG, KA, and DY: performed experiments. MG, KA, DS, JB, and BZ: analyzed data. KA, MG, and BZ: wrote the manuscript. AB, JD, and BZ: contributed to conception and design of the study.

## Funding

This work was supported by the National Heart, Lung, and Blood Institute (NHLBI) grant R01HL151740 (BZ and AB), the St. Baldrick's Foundation for Childhood Cancer (Award ID 638335, BZ and AB); and the National Institutes of Health's National Center for Advancing Translational Sciences, grant UL1TR002494 (BZ). AB was funded by a Minnesota Partnership for Biotechnology and Medical Genomics #18.04. JD is a Canada Research Chair in Molecular Medicine and is funded by a Foundation Grant awarded by the Canadian Institutes for Health Research (CIHR) and by a Women and Children's Health Research Institute (WCHRI) grant, University of Alberta.

## Author Disclaimer

The content is solely the responsibility of the authors and does not necessarily represent the official views of the National Institutes of Health.

## Conflict of Interest

The authors declare that the research was conducted in the absence of any commercial or financial relationships that could be construed as a potential conflict of interest.

## Publisher's Note

All claims expressed in this article are solely those of the authors and do not necessarily represent those of their affiliated organizations, or those of the publisher, the editors and the reviewers. Any product that may be evaluated in this article, or claim that may be made by its manufacturer, is not guaranteed or endorsed by the publisher.

## Abbreviations

ANGII: Angiotensin II
DOX: Doxorubicin
ISO: Isoproterenol.
