# Supplementary figures and images for: Divergent Cardiac Effects of Angiotensin II and Isoproterenol Following Juvenile Exposure to Doxorubicin
Source: Front Cardiovasc Med. 2022 Mar 25;9:742193. doi: 10.3389/fcvm.2022.742193 (PMC8990895; doi:10.3389/fcvm.2022.742193)

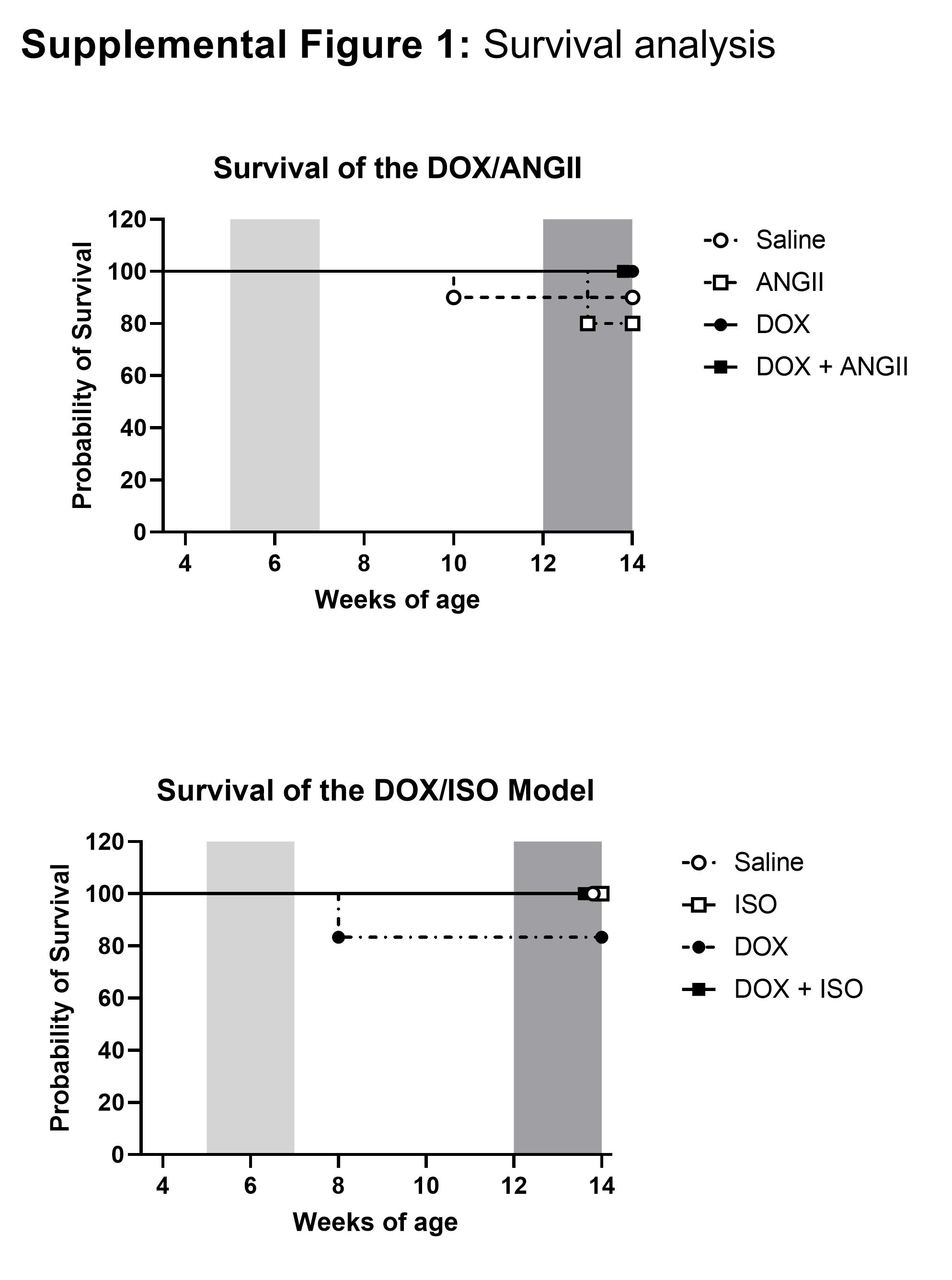

Supplement: Supplementary file 1 [file Image_1.tif]
