# Supplementary material for: Divergent Cardiac Effects of Angiotensin II and Isoproterenol Following Juvenile Exposure to Doxorubicin
Source: Front Cardiovasc Med. 2022 Mar 25;9:742193. doi: 10.3389/fcvm.2022.742193 (PMC8990895; doi:10.3389/fcvm.2022.742193)
Supplement: Supplementary file 2 [file Table_1.DOCX]

**Supplementary Table 1. Primer sequences used in this study.**

| **Gene** | **Forward primer (5’-3’)** | **Reverse primer (3’-5’)** |
| --- | --- | --- |
| *ANP* | GGAGCCTACGAAGATCCAGC | TCCAATCCTGTCAATCCTACCC |
| *BNP* | AGTCCTTCGGTCTCAAGGCA | CCGATCCGGTCTATCTTGTGC |
| *COX-2* | CTGGTGCCTGGTCTGATGATG | GGCAATGCGGTTCTGATACTG |
| *Collagen 1a1* | CTGGCGGTTCAGGTCCAAT | TTCCAGGCAATCCACGAGC |
| *Collagen 3a1* | ATGGTGGTTTTCAGTTCAGCTATG | GCCCGGCTGGAAAGAAGT |
| *Lgals-3* | TATCCTGCTGCTGGCCCTTATG | GTTTGCGTTGGGTTTCACTG |
| *Ace* | CAGTGTCTACCCCCAAGCAT | TTCCATCAAAGACCCTCCAG |
| *Agtr1a* | CAACTGCCTGAACCCTCTGT | TGTTATCTGAGGGGCGGTAG |
| *Agtr1b* | CAATTGCCTGAACCCTCTGT | TTATCTGAAGGGCGGTAGGA |
| *Beta-actin* | TATTGGCAACGAGCGGTTCC | GGCATAGAGGTCTTTACGGATGTC |
